# Expression and Regulation of Facilitative Glucose Transporters in Equine Insulin-Sensitive Tissue: From Physiology to Pathology

**DOI:** 10.1155/2014/409547

**Published:** 2014-03-04

**Authors:** Véronique A. Lacombe

**Affiliations:** Department of Physiology Sciences, Center of Veterinary Health Sciences, Oklahoma State University, Stillwater, OK 74078, USA

## Abstract

Glucose uptake is the rate-limiting step in glucose utilization in mammalians and is tightly regulated by a family of specialized proteins, called the facilitated glucose transporters (GLUTs/SLC2). GLUT4, the major isoform in insulin-responsive tissue, translocates from an intracellular pool to the cell surface and as such determines insulin-stimulated glucose uptake. However, despite intensive research over 50 years, the insulin-dependent and -independent pathways that mediate GLUT4 translocation are not fully elucidated in any species. Insulin resistance (IR) is one of the hallmarks of equine metabolic syndrome and is the most common metabolic predisposition for laminitis in horses. IR is characterized by the impaired ability of insulin to stimulate glucose disposal into insulin-sensitive tissues. Similar to other species, the functional capability of the insulin-responsive GLUTs is impaired in muscle and adipose tissue during IR in horses. However, the molecular mechanisms of altered glucose transport remain elusive in all species, and there is still much to learn about the physiological and pathophysiological functions of the GLUT family members, especially in regard to class III. Since GLUTs are key regulators of whole-body glucose homeostasis, they have received considerable attention as potential therapeutic targets to treat metabolic disorders in human and equine patients.

## 1. Regulation of Glucose Transport in Healthy State

Glucose is of the most abundant and essential energy sources for both plants and animals, existing in various polymerized forms such as cellulose and glycogen [1]. Glucose uptake from the bloodstream into the cell is the rate-limiting step in glucose utilization primarily in insulin-sensitive tissue in all species. Striated (i.e., cardiac and skeletal) muscle is the main tissue to utilize glucose as an energy substrate, followed by adipose tissue. For instance, skeletal muscle, which makes up ~40% of the body mass in mammalian species, is the primary tissue responsible for the peripheral disposal of glucose, especially during exercise [2]. In addition, the energetic demands in the heart are extreme and as a result, the heart has the highest rate of oxygen consumption per gram of any tissue in the body [3]. In order to sustain this high energy demand, the rate of glucose utilization in the heart is greater than in skeletal muscle, adipose tissue, and lung, despite the ability of the myocardium to use other substrates (i.e., fatty acids, lactate, ketone bodies, and amino acids) [4]. Therefore, glucose transport and utilization by myocytes are critical for the maintenance of muscle function [5]. In addition, glucose absorbed from the gut stimulates the release of insulin from pancreatic *β*-cells, which both suppresses hepatic gluconeogenesis and promotes glucose uptake by muscle and adipose tissue [6]. Finally, glucose can regulate gene transcription, epigenetic processes, and enzyme activity, as well as glucose-sensitive neurons in the brain [1].

In all mammalian cells, blood glucose is maintained within a narrow range by homeostatic mechanisms. Since the lipid bilayers of cell membranes are impermeable to glucose, most cells take up glucose by a passive facilitative transport process, which is mediated by a family of integral membrane proteins collectively known as the glucose transporters (GLUTs) [7]. It is believed that only the epithelial cell brush border of the small intestine and the kidney proximal convoluted tubules absorb glucose by an active mechanism that uses Na^+^/glucose cotransporters to transport glucose against its electrochemical gradient [8]. The equine intestinal Na^+^/glucose cotransporter, SGLT1, has 85–89% homology at the nucleotide level and 84–87% at the amino acid level with SGLT1 of other species [9]. Similarly to other herbivorous and omnivorous species, there is an enhancement in the expression of the intestinal Na^+^/glucose cotransporter, SGLT1, and the capacity to absorb monosaccharides in response to increased dietary carbohydrate levels in horses [10].

This paper reviews the current knowledge on the physiological and pathophysiological functions of the GLUT/SLC2 family member, as they relate to the equine species focusing on insulin-responsive tissue (e.g., striated muscle and adipose tissue).

### 1.1. Function and Distribution of the Facilitative Glucose Transporters

The GLUT protein family are encoded by the SLC2 genes and are a member of the major facilitator superfamily of membrane transporters, which are ubiquitous proteins with >5,000 members identified in all three kingdoms [1, 11]. Since at least one GLUT isoform is present in every cell type of the body, GLUTs are widely recognized as key regulators of whole-body glucose homeostasis. The GLUT proteins, which are comprised of ~500 amino acid residues, have 12 membrane-spanning domains, an N-linked glycosylation site, and intracellular NH_2_ and COOH termini, as well as several conserved residues and motifs designated “sugar transporter signatures” [7, 12]. These facilitative GLUT proteins are expressed in a tissue-specific manner, with distinct regulatory and/or kinetic properties that reflect their specific roles in cellular and whole-body glucose homeostasis [1]. Currently, fourteen GLUT isoforms have been identified and divided into 3 different classes based on sequence similarity, as well as structural and functional characteristics: Class I (GLUTs 1–4, 14); Class II (GLUTs 5, 7, 9, and 11), and Class III (GLUTs 6, 8, 10, and 12 and HMIT). Although the major substrate(s) for several isoforms has not been identified, these facilitated transporters are capable of transporting glucose, fructose, myoinositol, and urate across the plasma membrane down-gradient. Tissue distribution and major function(s) are listed in Table 1. Since several reviews on the GLUT family have been published [1, 6–8, 13], this paper briefly summarizes key features of the major GLUTs, as they relate to the equine species. The function and distribution of two members of class I, GLUT-1 and -4, have been extensively studied [1, 14, 15]. GLUT1 is primarily located at the cell surface and thus is a basal transporter that does not require insulin stimulation for its activation. Therefore, GLUT1 is considered to be the primary transporter responsible for basal glucose uptake in many cell types. GLUT1 has a high expression in brain and erythrocytes [16], and as a consequence, ~1/3 of blood glucose is carried within the red blood cell cytoplasm [7]. Interestingly, GLUT1 is also highly abundant in the digital lamellae, indicating that this unique weight-bearing, dermoepidermal structure within the horse's hoof is a highly metabolic tissue [17, 18]. Similarly, articular chondrocytes, which express both GLUT-1 and -3, rely on glucose as the major energy substrate and as the main precursor for the synthesis of extracellular matrix glycosaminoglycans in cartilage [19, 20]. The equine small intestine is the major site of glucose absorption, with glucose transport being the highest in the duodenum, followed by the jejunum and ileum [9]. Glucose is transported across the brush-border membrane of the intestinal enterocytes by SGLT1 and then exits the cell across the basolateral membrane by the facilitated GLUT2, a low-affinity glucose and fructose transporter [10, 21]. Equine small intestine also expresses GLUT5, a fructose transporter, in the villus enterocytes with the highest levels in the duodenum and the lowest levels in the ileum [22].

In contrast to basal GLUTs, GLUT4 translocates from an intracellular (nonactive) pool to the cell surface (active site) to enhance glucose uptake, a process called translocation. Therefore, GLUT4 is responsible for insulin-stimulated glucose uptake [23, 24]. Since GLUT4 is predominantly expressed in insulin-sensitive tissues, it plays a crucial role in whole-body glucose homeostasis [24] and as a result is one of the most studied GLUT isoforms, including horses. The equine GLUT4 gene has been characterized in equine skeletal muscle, which shares a high degree of homology with that of other mammalians [17, 25–27]. Similar to other species, GLUT4 protein is abundantly expressed in striated muscle and adipose tissue of horses [27, 28]. In addition, GLUT4 protein is expressed in equine muscle in a fiber-type specific manner with a higher expression in type 2B fibers, compared with that in type 1 fibers [27]. In contrast, immunostaining of GLUT4 in the equine digital lamellae has produced inconclusive results [29, 30], while western blot of crude membrane and PCR showed that GLUT4 is not a predominant isoform, suggesting that this insulin-responsive GLUT is unlikely to play a substantial role in the regulation of lamellar glucose transport [17, 18]. In addition, glucose uptake was not affected by insulin, further indicating the insulin-independent nature of the equine lamellae [17].

Although it has been believed that GLUT4 translocation is the rate-limiting step for glucose uptake and utilization in insulin-sensitive tissue, GLUT4 knockout mice do not develop hyperglycemia [31], suggesting that other GLUT isoforms could be involved in the regulation of whole-body glucose homeostasis [28]. Therefore, researchers have recently focused their attention on less understood GLUTs, particularly class III transporters, such as GLUT-8 and -12 [12, 32–35]. For instance, GLUT12 has been identified as a potential novel second insulin-sensitive GLUT [36], although its function is more likely to be tissue specific as for many other isoforms. In addition, its role is not well known in any species [37]. In horses, GLUT12 is expressed in insulin-sensitive tissues, with the highest protein expression in the omental adipose tissue, similar to the distribution pattern of GLUT4 protein across tissues [28]. In addition, it has been suggested that GLUT8, a dual-specific glucose and fructose transporter, is another insulin-regulated GLUT because it is highly expressed in striated muscle and adipose tissue, in addition to other tissues such as brain, testis, spermatozoa, liver, and kidney [38]. Furthermore, GLUT8 has been identified as an insulin-responsive GLUT in blastocysts [39]. Interestingly, GLUT8 protein has been recently characterized in striated muscle and digital lamellae of horses, with similar protein expression across tissue [18]. However, further studies are required to elucidate the physiological functions of the novel GLUT-8 and -12 isoforms.

### 1.2. Regulation of GLUT Translocation

**Figure 1:**
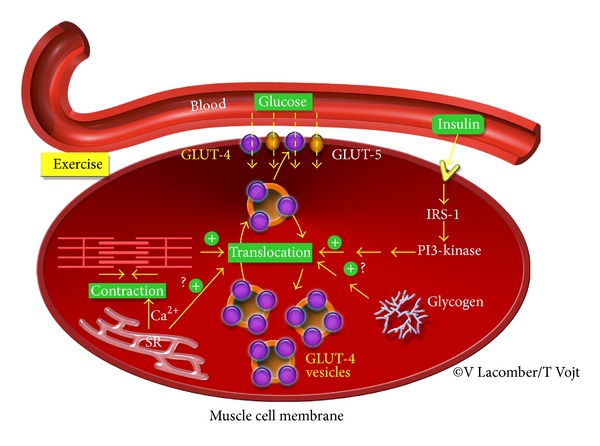
Regulation of glucose transport in striated muscle. GLUT4 translocation to the cell surface is mediated by both insulin-dependent and -independent pathways. GLUT: glucose transporter; PI 3-kinase: phosphatidylinositide 3-kinase; IRS: insulin receptor substrate; Ca^2+^: calcium; SR: sarcoplasmic reticulum.

Despite intensive research for over 50 years (primarily in rodents), the downstream signaling pathways that mediate the translocation of GLUT4 protein from an intracellular pool to the sarcolemma (active site) are not fully elucidated in any species. GLUT4 trafficking is largely regulated by insulin-dependent processes, although other factors including calcium/contraction, catecholamines, and hypoxia can alter glucose transport in skeletal and cardiac muscles (Figure 1) [40–47]. Since there are limited reports on the equine species, most of the information presented in this section is derived from studies on humans and rodents.

**Figure 2:**
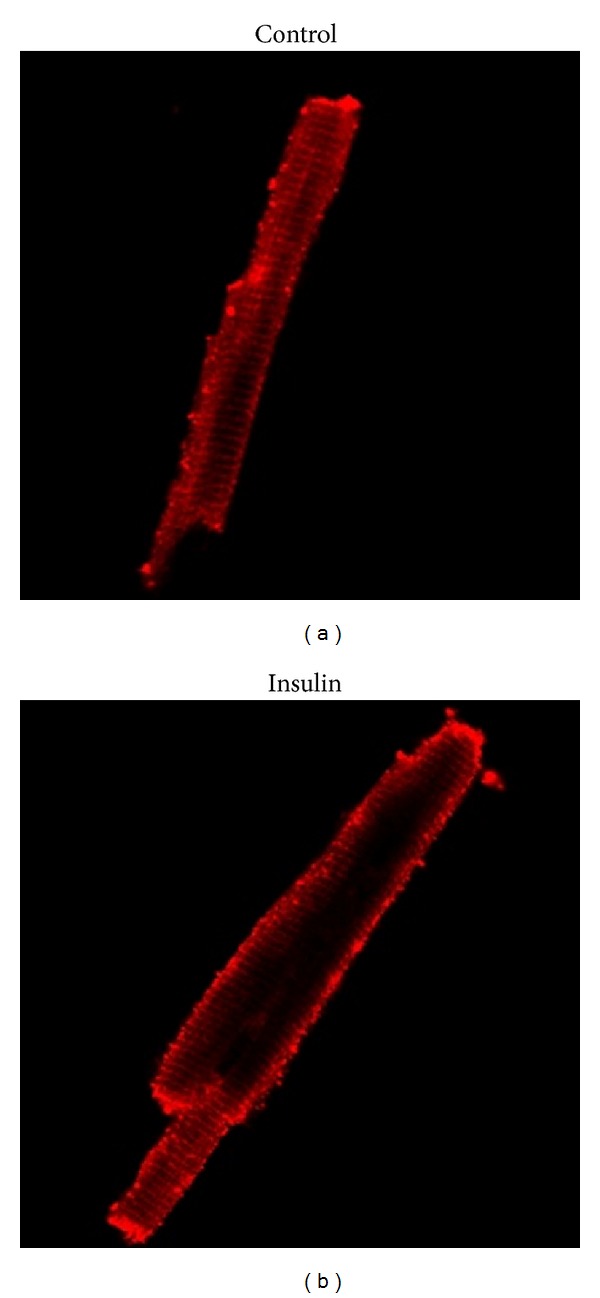
Insulin translocates GLUT4 from an intracellular pool (inactive site) to the cell surface (active site) in muscle, as visualized by confocal laser scanning microscopy. Adult rat cardiac myocytes were incubated without (a) or with insulin ((b), 100 *μ*U/mL) for 30 min prior to immunofluorescent staining. Modified from [37].

GLUT4 is a continuously recycling protein with faster endocytosis than reexocytosis, resulting in a net, but dynamic, intracellular retention [48]. The concept that insulin mobilized GLUT4 from intracellular storage vesicles to the plasma membrane was proposed by Cushman and colleagues in 1980 [49]. It has been estimated that ~5% of total GLUT4 is located at the cell surface of myocytes and adipocytes during basal conditions and that this fraction increased to ~50% after insulin stimulation (Figure 2) [6]. However, there is still some considerable debate on the regulation of GLUT trafficking by the downstream insulin signaling pathways [6]. It is known that, upon insulin release, insulin binds to the insulin receptor, thereby triggering its intrinsic protein-tyrosine kinase activity. The subsequent autophosphorylation of several insulin receptor tyrosine residues promotes tyrosine phosphorylation of insulin receptor substrates [50]. That action is followed by the activation of phosphatidylinositide 3-kinase (PI 3-kinase) and the production of phosphatidylinositol 3,4,5 triphosphate (PIP3) in the membrane (Figure 1). Increased PIP3 levels activate PDK1, a serine/threonine kinase, which in turn recruits the pivotal serine/threonine protein kinase, namely, Akt. Since it is involved in many processes, including cellular growth, survival, and fuel metabolism, Akt has 3 isoforms that directly act on several substrates [7]. Importantly, Akt2 appears to regulate AS160 (namely, Akt substrate protein of 160 kDa), which is the most distal signaling protein that has been implicated in insulin-mediated GLUT4 translocation and as such has emerged as a key regulator of GLUT trafficking. Under basal conditions, AS160 retains GLUT4 vesicles intracellularly through activity of its GTPase activating protein (GAP) domain for members of the Rab G protein family. The Rab proteins, which are guanosine triphosphate (GTP)-binding proteins, are thought to promote vesicle traffic (formation, targeting, and fusion) and may act as molecular switches, catalyzing membrane trafficking events by conversion from an inactive guanosine diphosphate-bound form to an active GTP-bound form [48]. Phosphorylation of AS160, which occurs on at least 6 different phospho-Akt-substrate (PAS) sites, suppresses or alters GAP activity, thereby elevating the GTP form of Rab, so GLUT4 exocytosis is permitted [51–53]. Although AS160 is also expressed in insulin-sensitive tissue of horses, its role in regulating GLUT trafficking has not been established [28, 54]. Finally, it has been shown that insulin-dependent GLUT4 translocation also requires dynamic remodeling of filamentous actin [48, 55]. Recently, it has also been suggested that *α*3(V) collagen is critical for glucose homeostasis, as mice with null alleles of the *α*3(V) gene Col5a3 have defective GLUT4 translocation to the plasma membrane in response to insulin [56].

Since exercise is vital for the maintenance of glucose homeostasis in diabetic patients, it has been proposed that insulin-independent pathways could be a major mechanism to regulate glucose uptake into striated muscles. Indeed, GLUT4 translocation to the cell surface can be regulated by contraction/Ca^2+^-dependent processes in striated muscle (Figure 1). For instance, contractile activity during exercise increases glucose uptake and the number of GLUTs at the cell surface of the skeletal muscle, in the absence of insulin [40–42, 57, 58]. Holloszy and Narahara, in 1967, suggested the possibility that exercise-induced contraction and the resultant increase in cytosolic Ca^2+^ provide the signal to activate glucose transport in frog skeletal muscle [59]. In addition, exposing skeletal muscle to caffeine, which induces Ca^2+^ release from the sarcoplasmic reticulum at a concentration too low to induce contractions, results in GLUT4 translocation, suggesting that intracellular Ca^2+^ spikes are sufficient to stimulate glucose transport [60]. However, since the seminal discovery in 1967, the molecular signaling mechanisms by which contraction/Ca^2+^ regulates muscle glucose transport have remained elusive in all species. It has been hypothesized that activated PI3-kinase directly or indirectly opens Ca^2+^ channels. In striated muscle, Ca^2+^ entry subsequently initiates contraction via Ca^2+^-induced Ca^2+^ release through the ryanodine receptors, which releases Ca^2+^ from the sarcoplasmic reticulum and subsequently activates intracellular calmodulin and calmodulin-dependent protein kinase II (CAMPKII). However, the mechanisms by which calmodulin and CAMPKII regulate glucose transport are not well known [58, 61]. Alternatively, it has been suggested that Ca^2+^ plays a direct role in the insertion of GLUT4 at the sarcolemma. Another potential candidate for contraction-stimulated glucose transport in skeletal muscle is the protein kinase C family [62]. However, the role of these potential candidates has not been well established because of the lack of specificity of most inhibitors studied [62]. Finally, during exercise, ATP is hydrolyzed to sustain the cross bridge cycle and the activity of ion channels and pumps involved in the excitation-contraction process, such as the sarcoplasmic reticulum calcium ATPase (SERCA) pump. The subsequent increase in AMP/ATP ratio has been implicated as a mediator of contraction-induced GLUT4 translocation in skeletal muscle, through activation of AMP-activated protein kinase (AMPK) [62]. There is recent evidence that activation of AMPK is involved in the regulation of AS160 through a contraction/Ca^2+^ dependent process in skeletal muscle. Indeed, AMPK phosphorylates AS160 at a site (namely, S711), which is distinct from phosphorylation sites that are modulated by insulin downstream signaling pathway [52, 63]. Although it has been shown that high-intensity exercise increases GLUT4 content in skeletal muscle of horses [64], further exploration of the molecular regulations of GLUT4 trafficking by insulin-independent pathways has not been established in this species. Only one study reported an increase in plasma AICAR (5-amino-4-imidazolecarboxamide riboside) after exercise in race horses [65]. Since AICAR activates AMPK, one could speculate that, similar to others species, AMPK-mediated metabolic regulation could be an important process for exercise-induced increased glucose transport in horses. Therefore, additional studies are needed to further understand the mechanisms of insulin-independent glucose transport.

### 1.3. Contribution of Glucose Transport to Muscle Glycogen Synthesis and Whole-Body Insulin Sensitivity in Healthy Horses

**Figure 3:**
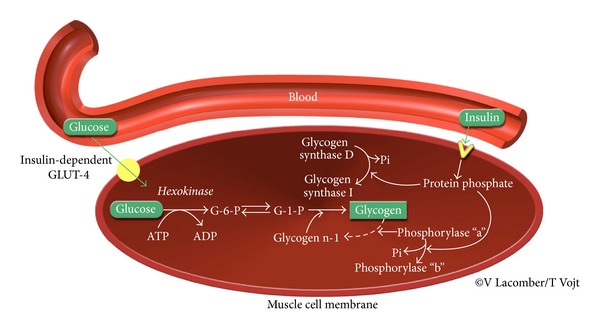
Biochemical pathways underlying glucose uptake and glycogen synthesis in the skeletal muscle after a high soluble carbohydrate diet. Insulin (1) activates GLUT4 translocation to enhance glucose uptake; (2) activates protein phosphatase, which converts glycogen synthase from its inactive form (D) to its active form (I); and (3) inhibits glycogenolytic enzymes such as phosphorylase a. G-6-P: glucose-6-phosphate; G-1-P: glucose-1-phosphate. Modified from [47].

Similar to humans, horses rely on carbohydrate metabolism during both high- and low-to-moderate intensity exercise, where glycogen and glucose are the obligate substrates to sustain anaerobic ATP production [66]. Therefore, during exercise, the majority of circulating glucose is utilized by muscle fibers to sustain contraction. At rest, the majority of the glucose entering the cell is converted to triglycerides in adipose tissue and to glycogen in muscle fibers and liver. Therefore, the capacity to maximize muscle glycogen replenishment after exercise is an important factor for optimizing subsequent performance in horses [47]. After exercise, the feeding of a high soluble carbohydrate diet (such as grains and sweet feeds) hastens glycogen synthesis by increasing glucose availability and insulin release. Similar to other species, insulin release activates protein phosphatase, which converts glycogen synthase from its inactive form (D) to its active form (I) and inhibits glycogenolytic enzymes such as phosphorylase a (Figure 3). Since glucose is the required substrate for glycogen synthesis, insulin release also activates GLUT4 translocation to the plasma membrane to enhance glucose uptake [47, 50]. However, in contrast to other species, in which complete synthesis of the muscle glycogen pool takes 24 h, it requires 48–72 h after exhaustive exercise in horses, even after feeding a high soluble carbohydrate diet [67]. It has been proposed that the mechanisms underlying the slow rate of glycogen replenishment after exercise in horses may include limited ability for the small intestine to digest starch and slower activity of the muscle glycogen synthase enzyme compared with values reported in humans and rodents [68, 69]. In addition, there is evidence that glucose transport across the sarcolemma is the rate-limiting step for glucose uptake and subsequent glycogen synthesis in equine skeletal muscle [47, 68]. For instance, intravenous glucose infusion after exercise enhanced muscle glycogen synthesis but failed to enhance protein expression of the insulin-responsive GLUT4 in muscle 24 hours after a strenuous exercise [64]. Similarly, starch-rich meals failed to enhance GLUT4 gene expression, GLUT4 protein content, and muscle glycogen concentrations within the first 24 h after strenuous exercise [67, 70]. Finally, it was reported that *in vitro* insulin stimulation minimally increased GLUT4 translocation in the equine skeletal muscle, with the increase only being ~15% even after supraphysiological insulin concentrations in healthy ponies [54, 71]. These changes are much less than the ones reported in skeletal muscle of healthy human after physiological (80%) and supra-physiological (400%) insulin stimulation [71, 72]. To further evaluate *in vivo* glucose transport in horses, researchers used the euglycemic hyperinsulinemic clamp technique since the induced supraphysiological plasma insulin concentrations produce a maximal response of glucose uptake in insulin-sensitive tissue while inhibiting endogenous hepatic glucose production [2]. Interestingly, both short-term (i.e., 6 h) and long-term (i.e., 48 h) exogenous hyperinsulinemia in healthy horses did not affect the expression of GLUT4 gene and protein in equine skeletal muscle [18, 73]. Therefore, the observed lack of substantial increase in GLUT4 expression and/or translocation in healthy skeletal muscle in response to both *in vivo* and *in vitro* insulin stimulation may be the result of the insulin-dependent signaling pathways regulating GLUTs already being at near maximal physiologic limits for the species [54]. Alternatively, it has also been suggested that the relatively high resting muscle glycogen concentrations typical of the equine species may partially prevent further GLUT4 translocation to the plasma membrane via a negative feedback pathway [64]. Taken together, these studies demonstrated that glucose uptake across the sarcolemma is the rate-limiting step in glucose utilization and could be a major mechanism for the slow rate of muscle glycogen replenishment observed after exercise in this species [50].

The central role of adipose tissue glucose transport in regulating whole-body insulin sensitivity is becoming increasingly evident in mammalians, as adipose tissue-selective reduction of GLUT4 in mice results in impaired glucose tolerance and peripheral IR [74]. In addition, human omental adipocytes have higher GLUT4 expression and basal- and insulin-stimulated glucose uptake rate [75]. Similar to humans, GLUT4 protein expression is higher in visceral compared to subcutaneous adipose sites and skeletal muscle of insulin-sensitive horses, with the highest content present in the omental site [28]. Since GLUT4 is abundant in visceral adipose tissue, it is therefore likely to play a substantial role in the regulation of visceral glucose transport and in the maintenance of peripheral insulin sensitivity. Overall, the greater ability of insulin to promote glucose uptake in visceral compared with subcutaneous fat might be explained by higher levels of insulin receptors and downstream intermediates in the former fat depot, which could also explain the higher insulin-responsive GLUT content in this depot [76]. In support of these findings, GLUT12, a novel potential second insulin-responsive GLUT, has a higher protein content in visceral compared to subcutaneous adipose tissue and skeletal muscle in healthy horses [28]. Collectively, these findings suggest a central role for visceral GLUT-mediated glucose transport in the maintenance of peripheral insulin sensitivity in healthy horses.

## 2. Glucose Transporters: Implications for Metabolic Dysregulation

Although increased insulin sensitivity has been reported with equine polysaccharide storage myopathy and equine motor neuron disease, changes in GLUT expression and/or trafficking have not been reported [21, 77]. In contrast, impaired glucose uptake appears to play a key pathogenic role during insulin resistance (IR) and other related metabolic diseases in many species, including horses, as outlined below.

### 2.1. Dysregulation of Glucose Transport: Parallels between Equine Metabolic Syndrome and Type 2 Diabetes in Humans

There is an emerging and growing threat as well as considerable health issues associated with insulin resistance, diabetes, and obesity, which are primary health concerns for humans and affect a wide range of economically important domesticated and/or agricultural species [78–81]. In humans, the World Health Organization has declared diabetes an epidemic disease, with worldwide prevalence expected to rise to 438 million by 2030 and projects that the number of diabetes-related deaths will increase by more than 50% worldwide in the next 10 years [82]. Prior to the development of type 2 diabetes, patients almost always suffer from “prediabetes,” which is characterized by impaired glucose tolerance, increased fasting glucose (with values above the normal range but below the cutoff for the diagnosis of diabetes, 100 to 125 mg/dL), or both [83]. Development of diabetes from normal glucose tolerance is a continuous process, which may last for over a decade. Over 300 million people suffer from this preclinical stage of diabetes and this figure is expected to rise to 472 million worldwide by 2030 [83]. Insulin resistance is a core component of the preclinical stages of type 2 diabetes (i.e., “prediabetes”) and is characterized by an impaired ability of insulin to stimulate glucose uptake into muscle and adipose tissue in all species. Insulin resistance first leads to compensatory oversecretion of insulin by the pancreatic *β*-cells and, eventually, *β*-cell exhaustion and development of type 2 diabetes. In addition, IR in the liver results in failure to suppress glucose production and release into the blood, further worsening the hyperglycemia. Although the development of diabetes is rarely reported in horses [84], there have been increased clinical awareness and recognition of chronic IR without marked hyperglycemia [85–88]. In addition, equine metabolic syndrome phenotype has been defined as a general clustering of obesity, IR, hyperinsulinemia, hypertriglyceridemia, and an increased risk for laminitis [85–88]. Affected patients are adult horses of both genders, from a wide range of breeds, types, and performance levels, living in distinct parts of the country [85, 88–90]. With increasing awareness, there is increasing diagnosis. Although the true prevalence of IR is not known, it has been estimated to be 10–28% (based on hyperinsulinemia) when large populations of adult horses have been studied [91]. It is anticipated that the prevalence of animals at metabolic risk of developing IR is even greater. Although there is limited work on biomarkers or panels of risk factors, environmental and physical risk factors for equine IR have been identified. Diet appears to be one important factor, as significant parallels exist between the provision of refined, nutritionally dense rations to horses and the consumption of energy dense refined foods by people in modern society [92]. In addition, increasing age, pregnancy/maternal insulin sensitivity, and obesity are also associated with equine IR [92–96]. As in other domesticated animals (e.g., dogs and cats), obesity is prevalent in horses and reported to be 19% and 32% in two independent studies of at least 300 adult horses [86]. Similar to humans, obesity promotes IR in horses, although IR occurs in nonobese equines as well [95, 96].

Although there has been increased clinical recognition of IR and diabetes, a clear understanding of the pathophysiology of these insidious diseases is still missing in horses and in other species. Cellular defects associated with IR can include reduced insulin receptor number, reduced expression and activation of insulin signaling cascade proteins (e.g., PI3K), and an impaired glucose transport process [54, 97]. Importantly, alterations in GLUT4 translocation appear to be a key pathogenic factor underlying IR in multiple species, including in horses. For example, decreased insulin-stimulated GLUT4 translocation in skeletal muscle is a hallmark of IR associated with type 2 diabetes in humans and rodents [98, 99]. Although there have been limited studies in horses, it was recently demonstrated that naturally occurring IR decreases translocation of GLUT4 to the cell surface in equine skeletal muscle, without a change in its total protein expression [54]. It is worthy to note that these horses exhibited early stage IR (with normal baseline blood glucose concentrations), suggesting that the observed alteration in muscle glucose transport is an early pathogenic event [54].

In many species, it is becoming increasingly apparent that adipose tissue plays a key pathogenic role in the overall development of IR [74]. Chronic elevation of serum free fatty acid concentrations, as observed in many humans with obesity or diabetes and horses with Cushing's or metabolic syndrome, may also contribute to the decreased uptake of glucose into peripheral tissues. It is well accepted that preferential deposition of fat into visceral deposits instead of subcutaneous deposits increases the risk of IR and diabetes in humans [100]. The “Portal Theory” suggests that IR and many of its related features could arise from visceral adipose tissue delivering free fatty acids at a high rate to the liver via the portal vein into which visceral adipose tissue directly drains. This in turn increases hepatic glucose production, reduces hepatic insulin clearance, and ultimately leads to hyperinsulinemia and IR [50]. In addition, the development of obesity-induced IR may be caused by visceral fat being more sensitive to increase in adipocyte cell size during expansion of adiposity, which may result in a relative decrease in cellular GLUT4 content as cell size increases [28, 50]. In contrast, in horses, adiposity and expansion of fat deposits in the crest and tailhead regions are associated with IR [50]. However, the importance of these regional fat sites to the pathogenesis of IR and glucose transport in horses has not been well investigated. One study reported a decrease in GLUT4 translocation in both visceral and subcutaneous adipose tissue in insulin-resistant compared to insulin-sensitive horses, suggesting an impairment of glucose transport in both of these adipose tissues [28]. Similarly, GLUT4 protein expression was lower in visceral adipose tissue of type 2 diabetic humans [101] and to a lesser extent in subcutaneous adipose tissue [102].

Since AS160 is a downstream insulin signaling protein, it could be a potential mechanism for the observed alteration in GLUT4 translocation to the cell surface during metabolic diseases. However, IR did not induce alterations in total nor phosphorylated AS160 in skeletal and adipose tissue of horses, suggesting that alterations in glucose transport occurred despite normal AS160 activation [28, 54]. Similarly, the role of AS160 in the pathogenesis of IR has not been well established in other species [6].

Overall, the early pathophysiological mechanisms underlying the long latency period preceding overt diabetes remain largely undefined in humans. Interestingly, horses are frequently affected by chronic IR and rarely decompensate to diabetes. As such, this species could provide a unique naturally occurring animal model to elucidate the chronic, pathological changes accompanying the transition from normal to impaired glucose tolerance. However, it should be pointed out that, because of its evolution as a grazing, hindgut-fermenting herbivore, the horse presents some unique features in regard to its metabolism [103]. For example, the horse derives ~30% of its energy from volatile fatty acids produced in the cecum, and acetate (the main volatile fatty acid produced in the hindgut) could account for 30% of the energy utilization by the limb of healthy horses during periods of rest [104–106]. Nonetheless, horses with chronic IR present some similarities in regard to metabolic dysregulation with human patients affected by the preclinical stages of type 2 diabetes, both at the whole tissue and molecular levels.

### 2.2. Dysregulated Glucose Transport and Inflammation: Potential Novel Link and Pathogenic Factor

Numerous studies in humans and animal models of metabolic diseases have demonstrated that obesity induces an inflammatory state, which plays a key pathogenic role during IR [107, 108]. With the development of obesity, adipose tissue is reported in humans and rodents to adopt a distinct inflammatory phenotype, characterized by increased gene expression and secretion of proinflammatory cytokines, such as tumor necrosis factor (TNF-*α*) and interleukins (IL)-1 and -6 [108]. In response to the increased proinflammatory cytokines (i.e., TNF-*α*), there is an increased production of suppressors of cytokine signaling (SOCS) proteins, which have been suggested to play a crucial link between inflammation and IR in the liver [109–111]. However, the feedback regulation of SOCS-3 is complex, and there are conflicting reports describing its relation with other components of the inflammatory signaling pathways, including toll-like receptors (TLR) [112], and ultimately the pathways linking inflammation to altered glucose transport remain elusive in all species [113].

It is well known that intravenous administration of endotoxin, which activates TNF-*α* and TLR, impairs insulin sensitivity in horses [114]. However, the role of inflammation during IR in horses has received scarce attention and findings remain controversial. It was reported that peripheral blood cells of obese hyperinsulinemic horses showed decreased endogenous proinflammatory cytokine gene expression [115]. In contrast, a correlative relationship was demonstrated between biomarkers of inflammation in the blood (i.e., TNF-*α* and IL-1) and obesity and IR in horses, as well as laminitis [116–118]. Dr. Burns and colleagues reported that subcutaneous depot (nuchal ligament) might be more likely to adopt an inflammatory phenotype than other adipose depots [119]. Furthermore, a significant increase in TNF-*α* protein expression in visceral but not subcutaneous tissue was reported in horses with compensated IR, suggesting a role for inflammation during IR [120].

Recent studies suggested that toll-like receptor (TLR) could be a key mechanism by which impaired fatty acid metabolism induces and propagates inflammation, and ultimately decreases insulin signaling and glucose transport [121]. TLRs recognize pathogen-associated molecular patterns and are of primary importance in the innate and adaptive immune system. TLR stimulation also leads to inflammation and cytokine production [121]. Although lipopolysaccharide is the key activator of TLR4 signaling, fatty acids have also been shown to be an endogenous ligand for TLR4 [122]. Activation of TLR4 signaling in adipocytes could activate JNK and NFkB signaling leading to increased production of proinflammatory cytokines, which in turn increases SOCS-3 production. Increased SOCS-3 in muscle and adipose tissue leads to decreased signal transduction through serine phosphorylation of IRS-1 and thus impairs the insulin signaling pathway, which ultimately results in dysregulation of glucose transport and homeostasis [122]. Therefore, TLR4, which is highly expressed in insulin-sensitive tissues, has been linked with the pathogenesis of IR [123, 124]. Recently, it has been shown that activation of TLR4, which is also highly expressed in striated muscle and adipose tissue of horses, was associated with an increased SOCS3 production during naturally occurring equine IR [120]. In addition, TLR4 signaling was activated in the digital lamellae of an experimental model of induced hyperinsulinemia, suggesting that inflammation contributes to the development of endocrinopathic laminitis [125]. Collectively, these findings suggest that TLR4 is a pathogenic marker during equine IR and/or metabolic dysfunction. However, further studies are required to elucidate the role of inflammation during dysregulated glucose metabolism in horses.

### 2.3. Complications Associated with Insulin Resistance: Potential Role of Dysregulated Glucose Transport

In humans, it is well accepted that IR even without overt diabetes can lead to multiorgan dysfunction and increased mortality [126, 127]. Evidence suggests that a similar state of inflammatory and multiple tissue complications occurs in insulin-resistant horses [85, 87]. For instance, IR is thought to be involved not only in the pathogenesis of equine metabolic syndrome and diabetes mellitus, but also in the pathogenesis of pars intermedia dysfunction, hyperlipemia, endotoxemia, osteoarthritis and osteochondrosis dissecans [19, 128]. Importantly, IR is well known to be a direct and independent risk factor for a painful, debilitating peripheral vascular limb disease in horses, namely, laminitis, with some similarities to peripheral vascular disease and ischemic necrosis of the foot in human diabetic patients [85, 87, 88]. The prognosis and outcomes for horses with laminitis are poor, varying from 8 to 20% dying or being euthanized after 1 year, and it has been suggested that hyperinsulinemic laminitis is ultimately not reversible [129]. Therefore, a better understanding of the early pathophysiological effects could improve our capacity to predict and prevent this debilitating disease. Although the proposed mechanisms involved in the pathogenesis of laminitis are numerous and interrelated, IR is at the forefront of current investigations [130–133]. To this end, the development of experimental models of hyperinsulinemia-induced laminitis in the horse has provided further insights into the pathogenesis of endocrinopathic laminitis, especially as it relates to the altered glucose metabolism [27, 131, 134]. This is particularly germane as the regulation of glucose transport in the lamellae in the healthy and laminitic horse remains scarce and controversial. Although GLUT1 has been consistently reported within lamellar keratinocytes, its expression during laminitis is variable [17, 18, 30]. An immunohistochemical study demonstrated loss of lamellar GLUT1 during chronic laminitis [29]. In contrast, lamellar GLUT1 gene and protein expression was unaffected in horses and ponies treated with a prolonged supraphysiologic infusion of insulin (which induces laminitis) suggesting that endocrinopathic laminitis is unlikely due to impaired glucose uptake and subsequent glucose deprivation in lamellae [17, 18]. Interestingly, it has been recently reported that both novel GLUT-8 and -12 (members of class III) proteins were upregulated in the equine lamellae following prolonged insulin infusion [18]. Interestingly, it has been recently suggested that GLUT8 mediates the deleterious metabolic effects associated with insulin resistance in mice fed a high-fructose diet [135]. However, it remains to be determined whether glucotoxicity contributes to the development of hyperinsulinemic laminitis in horses. In addition, it is not well established whether insulin resistance is required for the induction of endocrinopathic laminitis. Therefore, further studies are required to elucidate the pathogenesis of endocrinopathic laminitis, including additional potential mechanisms such as oxidative stress and vascular perturbations to the digit.

To further understand the impact of impaired glucose transport during diabetes, researchers have focused their attention on mechanisms by which chronic hyperglycemia induced micro- and macrovascular damages. Four main hypotheses have been proposed: (1) increased polyol pathway flux, (2) activation of protein kinase C isoforms, (3) increased hexosamine pathway flux, and (4) increased advanced glycation end products (AGEs) [136]. Importantly, chronic hyperglycemia accelerates the reaction between glucose and proteins and leads to the formation of advanced glycation end products, which form irreversible cross-links throughout the lifetime of many large proteins (such as collagen and hemoglobin), covalently modifying their structure and function [137–139]. In diabetes, and to a lesser extent during aging, AGEs accumulate at an accelerated rate in various cell types (in days to weeks) and cause multiple organ dysfunction [137, 139]. Once AGEs are formed, they are irreversible. At the organ level, AGEs stimulate collagen deposition, which ultimately leads to increased tissue fibrosis [137–141]. AGEs can also initiate alterations in cellular function by binding to a specific receptor known as the receptor for advanced glycation end products (RAGE), which is found on the surface of many cells, including macrophages, epithelial and endothelial cells, and smooth muscle cells. Although there is evidence that RAGE activation could occur early during the pathophysiological process, the role of RAGE and AGEs during metabolic dysfunction in horses has received scarce attention, in contrast to the extensive studies performed in human and rodents. Recently, one study reported a modest accumulation of AGE in the lamellar tissue of horses after 48 h of hyperinsulinemia-induced laminitis, suggesting a potential pathogenic role [142].

## 3. Glucose Transporters: Potential Therapeutic Targets

Since GLUTs are key regulators of whole-body glucose homeostasis during physiologic and pathophysiologic states, they have received considerable attention as potential therapeutic targets to treat metabolic disorders in humans, such as during diabetes and IR. However, despite the increasing clinical awareness, research on new pharmacologic approaches for equine IR is limited and effective insulin-sensitizing strategies are lacking.

As in humans, careful dietary management and increasing physical activity are suggested in horses to promote insulin sensitivity [86]. However, the difficulty of encouraging exercise and weight loss in a recurrently laminitic horse can make the management very challenging or even be contraindicated. Therefore, there is a need for pharmacological intervention in horses, and levothyroxine sodium (i.e., T4) has been prescribed to induce weight loss and to improve insulin sensitivity [143]. In other species, it has been shown that thyroid hormone increases glucose uptake in insulin-sensitive tissue, in part by the activation of GLUTs [144]. In addition, veterinarians have increasingly prescribed the off-label use of human medications, such as metformin, a biguanide [145]. Although the main mechanism of action of metformin is through the suppression of hepatic gluconeogenesis, its spectrum of action is wide and remains not completely understood [146]. Importantly, metformin activates AMPK, which induces GLUT4 translocation to the cell surface, and thereby increases insulin sensitivity by enhancing peripheral glucose uptake. However, no clinical effect was observed with oral doses of 11–16 mg/kg/day [147], and either no effect or only modest increases in insulin sensitivity were observed in insulin-resistant horses following 30 mg/kg/day oral metformin [145, 148]. In addition, long-term results appear to be variable, and possible adverse drug effects have been reported [147, 148]. This lack of prolonged therapeutic effect could be in part explained by the poor bioavailability of metformin in horses (36–52% lower than in humans) and thus, chronic dosing may not achieve therapeutic blood concentrations [146, 149]. Recently, pioglitazone, an FDA-approved thiazolidinedione to treat type 2 diabetes, has been evaluated in healthy horses. It is absorbed after oral administration without any clinically detectable adverse effects or any abnormalities in hematological and biochemical analyses [150]. Its mechanism of action relies on the activation of peroxisome proliferators-activated receptor gamma (i.e., PPAR-*γ* agonist). PPAR-*γ*, which is expressed in adipose tissue and muscle, is an intracellular transcription factor that controls the transcription of genes regulating glucose homeostasis [151, 152]. In particular, pioglitazone increases the efficiency of insulin-stimulated glucose uptake from blood into insulin-sensitive tissue by targeting GLUT proteins [153]. This increase in glucose uptake following pioglitazone treatment would in turn increase whole-body insulin sensitivity. In addition, pioglitazone possesses some anti-inflammatory properties including reduction of inflammatory markers (i.e., IL6, TNF-*α*), as well as prevention of SOCS-3 upregulation in the liver of insulin-resistant rodents fed a high fat/fructose diet [109], although similar effects have not been demonstrated in horses yet [154]. Pioglitazone could become an affordable therapeutic option for IR in horses since a generic preparation is going to be available soon [150]. However, only a few studies have investigated its therapeutic efficacy in horses. Although twelve days of pioglitazone treatment (1 mg/kg bodyweight PO) did not improve basal insulin-sensitivity, an increased skeletal muscle tissue transcript abundance of GLUT1 and insulin receptor was reported [155]. Therefore, further investigation of different dosing regimens of pioglitazone and metformin might be warranted in horses [146], as well as the investigation of alternative insulin-sensitizing strategies.

## 4. Conclusions

Glucose uptake is the rate-limiting step in whole-body glucose utilization and is tightly regulated by a family of specialized proteins, the glucose transporters. Insulin resistance is characterized by an impaired ability of insulin to stimulate glucose uptake into insulin sensitive tissues (i.e., muscle and adipose tissue) in all species. As for other species, the functional capability of the insulin-responsive glucose transporter GLUT4 is impaired in skeletal muscle and adipose tissue during naturally occurring IR in horses. Therefore, these insulin-responsive GLUTs provide molecular tool to further understand the pathophysiology of metabolic diseases across species and constitute potential therapeutic targets for equine patients affected by IR. However, despite intensive research for over 50 years, the molecular mechanisms of altered glucose transport remain elusive, and further research is required to characterize impairments of the downstream signaling pathways regulating GLUT4 translocation. Importantly, there is still much to learn about the physiological and pathophysiological functions of the GLUT family member, especially in regard to class III, and such knowledge will be essential in understanding the pathogenesis of IR and metabolic syndrome. Finally, future studies could also investigate novel physical, nutritional, and/or therapeutic strategies to activate GLUTs, which may be beneficial for both healthy equine athletes and horses with IR.

## Figures and Tables

**Table 1:** Summary of the main location and function of facilitative glucose transporter family [7, 8, 156].

Protein	Major sites of expression	Proposed function/major substrate	Major species studied
GLUT1	Many cell types (e.g., erythrocytes, brain); fetal tissues	Basal glucose uptake; transport across blood-tissue barriers	Rodents, humans, horses, cows, dogs, pigs
GLUT2	Kidney, small intestine, liver, pancreatic islets, brain	High-capacity low-affinity facilitated glucose and fructose transporter	Rodents, humans, horses, cows
GLUT3	Brain (neurons) and testis	High-affinity facilitated glucose transporter; neuronal transport	Rodents, humans, cows, dogs, pigs
GLUT4	Striated muscle, fat, heart	High-affinity facilitated glucose transporter; insulin-regulated transport; linked to IR/diabetes	Rodents, humans, horses, cows, dogs, pigs
GLUT5	Small intestine, kidney, striated muscle, fat, testis	Facilitated fructose transporter	Rodents, humans, horses, cows, dogs, pigs
GLUT6	Leukocytes, brain, spleen	Facilitated glucose transporter	
GLUT7	Small intestine, colon, testis	Transport of glucose and fructose	
GLUT8	Testis, brain, blastocyst, striated muscle, fat, liver, spleen, lung	Facilitated glucose transporter widely expressed; neuronal transport; insulin-responsive transport in blastocyst	Rodents, humans, cows, horses
GLUT9	Liver, kidney, small intestine	Facilitated urate (glucose) transporter	Rodents, humans
GLUT10	Striated muscle, lung, liver, pancreas		
GLUT11	Striated muscle	Muscle-specific; facilitated glucose/fructose transporter	Rodents, humans
GLUT12	Striated muscle, fat, prostate, mammary gland	A second insulin-responsive facilitated GLUT?	Rodents, humans, cows, horses
HMIT	Brain, fat	H^+^/myo-inositol cotransporter	
GLUT14	Testis	Orphan transporter	

## Abbreviations

GLUT: Glucose transporter
IR: Insulin resistance
PI 3-kinase: Phosphatidylinositide 3-kinase
PIP3: Phosphatidylinositol 3,4,5 trisphosphate
Ca^2+^: Calcium
AMPK: AMP-activated protein kinase
IRS: Insulin receptor substrate
AGE: Advanced glycation end products
TNF: Tumor necrosis factor
IL: Interleukins
TLR: Toll-like receptor
SOCS: Suppressors of cytokine signaling proteins
PPAR-*γ*: Peroxisome proliferators-activated receptor gamma
G-6-P: Glucose-6-phosphate
G-1-P: Glucose-1-phosphate
PDK1: Phosphoinositide-dependent kinase 1.
